# Nitrous Oxide

**Published:** 1868-09

**Authors:** 


*Nitrous Oxide.*—It is amusing to witness the excitement among our neighbors across the water in regard to this well-known anæsthetic. Experiment upon experiment has been tried and the surgeons are feeling their way carefully as though they were dealing with an exceedingly dangerous and deadly agent.

Dr. Richardson, in this matter, does not manifest that candour and devotion to scientific truth that we had a right to expect from so eminent an experimentalist. Having once pronounced against it, his pride of opinion is enlisted, and he perseveres in condemning it. He declares that it is not in itself, an anæsthetic, but that it acts by increasing the amount of carbonic acid in the blood, which excess produces the anæsthesia. Now this is well known to be the theory of many eminent observers concerning all anæsthetics. He continues to denounce it as dangerous, as indeed the most dangerous of all these agents. Such statements sound very strangely in this country, where it has been administered in innumerable cases, without a single untoward result, clearly traceable to the agent itself. It is another lamentable instance of the power of theory to blind the eyes to palpable facts. An additional characteristic of these discussions is that American experience is totally ignored. The Odontological Medical Society has appointed a committee to experiment and report.

